# Datasets of a Multizone Office Building under Different HVAC System Operation Scenarios

**DOI:** 10.1038/s41597-022-01858-6

**Published:** 2022-12-19

**Authors:** Yeobeom Yoon, Sungkyun Jung, Piljae Im, Anthony Gehl

**Affiliations:** Oak Ridge National Laboratory, One Bethel Valley Road, Oak Ridge, TN 37830 USA

**Keywords:** Energy modelling, Engineering

## Abstract

This study provides an open-source dataset of the measured weather data, building indoor data, and system data under the different test settings. The test building is the two-story Flexible Research Platform building at the US Department of Energy’s Oak Ridge National Laboratory, in Oak Ridge, Tennessee. Four heating tests and three cooling tests were conducted. The 1-min interval of weather, building indoor data, and system data from each test setting are provided. Actual weather data were collected from a weather station installed on the roof. This paper describes information on the test building and installed sensors, data collection method, and data validation. The provided dataset can be employed to understand HVAC system conditions and building indoor conditions under different HVAC system operations and the performance of building envelope without HVAC system operation using free-floating test data. Additionally, it can be used for empirical validation of the building energy modelling engine.

## Abstract (summary)

**Table:** 

Measurement(s)	temperature, humidity, energy, airflow rate, and solar radiation
Technology Type(s)	Data logger, building automation system, and weather station

## Background & Summary

In 2021, commercial buildings were one of the main consumers of energy, accounting for 18% and 35% of total energy consumption and total electricity energy consumption in the United States^1^. Specifically, 40% of energy is consumed by HVAC systems^2^. Because HVAC system energy consumption is the largest portion of commercial building energy consumption, there are many ongoing efforts to reduce building energy consumption by implementing a high-efficiency HVAC system (e.g., energy recovery ventilator, demand-controlled ventilation, variable refrigerant flow)^3–5^ and HVAC system controls (e.g., model predictive control, adaptive predictive control, pattern recognition adaptive control, and transactive control)^6–9^.

Since experimental testing is time-consuming and difficult to gather high-quality data from^10^, most case studies use building simulation models. To ensure the reliability of the simulation results, the building simulation model needs to be calibrated or validated before it is used. Because of minimal field data, calibrating all scenarios with field data is difficult. Therefore, the base model is typically calibrated with the field data, and a case study is performed based on the calibrated base model^11–13^. Alternatively, a reference building energy simulation model (e.g., prototype buildings developed by the US Department of Energy^14^) can be used for the case study^15–18^.

Existing open-source datasets related to building energy consumption fall under the following two main categories: residential buildings^19–21^ and commercial buildings^10,22,23^. For residential buildings, Jacoby *et al*. provided single-family household data related to indoor conditions with occupancy behaviour^19^. Makonin *et al*. provided single-family household data in Canada^20^. Schlemminger *et al*. provided data on 38 single-family houses relating to the heat pump system in a 1-min intervals^21^. For commercial buildings, Schweiker *et al*. provided 4 years of outdoor, indoor, and energy data for an office building in 10-min intervals^22^. Agee *et al*. provided 2 years of energy use data in 1-h intervals, energy production data from a renewable energy system in daily intervals, weather data in daily intervals, and building air leakage data^23^. Luo *et al*. provided building datasets including indoor data in 1 min intervals, outdoor data in 15-min intervals, energy data in 15-min intervals, system data in 1-min intervals, and occupant data in 10-min intervals under two test settings (i.e., conventional rule-based control and model-predictive control)^10^.

Most previous open-source datasets have focused on indoor and energy use data under one test setting and have considered occupied buildings. There are currently no open-source datasets generated based on different HVAC system operations in an unoccupied building showing the impact of the HVAC system operations on the building.

Because of the absent historical open-source datasets that focus on HVAC system operation, the goals of this paper are to provide detailed weather data, indoor conditions, and HVAC system data under different HVAC system operations in multizone commercial building, including constant thermostat setpoint and thermostat setback schedule. In general, multizone field tests have many uncertainties in monitoring data mainly due to uncertain and untracked occupancy behaviour and associated operating schedule of the building. As utilizing this unique facility, whose occupancy can be controlled (e.g., emulated), the dataset can have limited uncertainties among different HVAC operation scenario, which can provide apples-to-apples comparison among each case (except different weather condition).

This dataset has the following unique features:

- High quality, high-resolution one-minute interval data of the unoccupied multizone office building.
- To reduce the uncertainty and fair comparison among different operating scenarios, no internal heat gain (e.g., people, lighting, and equipment) was emulated.
- Dataset includes not only energy/power related data, but also indoor thermal condition data such as temperature, relative humidity per each zone.
- Dataset includes on-site detailed weather data including outdoor temperature, RH, wind, solar, etc.
- Provide the energy consumption data of each component in the installed HVAC system, including the compressors, condensers, supply fan, and variable air volume (VAV) boxes, and the airflow rates of the rooftop unit (RTU) and each VAV box.

The potential use cases for the dataset can be as followings:

- Understanding the performance of building envelope without HVAC system operation using free-floating test data.
- Develop a data driven or grey box model of the building to be used for advanced controls such as model predictive controls, and automatic fault detection and diagnosis (AFDD).
- Develop and train machine learning (ML) models to predict the building and system behaviour.
- Evaluate the weather impact on energy use for the target building – Performing sensitivity study on weather data.
- Use for calibration of the building energy simulation model.

As an example, Im *et al*. used cooling season dataset to validate the EnergyPlus simulation model^24^.

## Methods

### Facility information

**Fig. 1:**
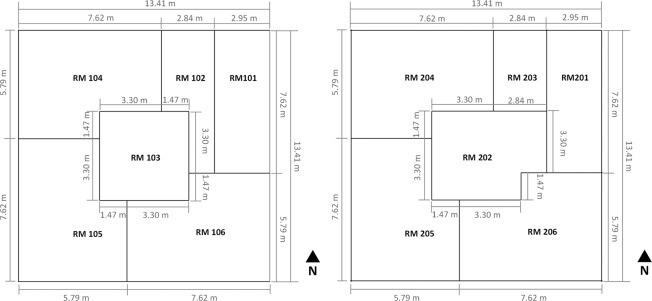
Floor plan of the FRP-2 building.

The two-story Flexible Research Platform (FRP-2) at the US Department of Energy’s Oak Ridge National Laboratory in Oak Ridge, Tennessee was selected as the test building. The building’s width and length are both 13.41 m, as shown in Fig. 1. Each floor has 5 conditioned zones (for a total of 10 conditioned zones), and an unconditioned staircase connects the floors. Floor to floor height of the first floor is 4.1 m, and floor to ceiling height is 2.7 m. On the second floor, floor to floor height is 4.2 m, and floor to ceiling height is 2.4 m. Table 1 lists the building construction information. The window to wall ratio of the FRP-2 building is 28%. No internal heat gain (e.g., people, lighting, equipment) was considered during the test to avoid uncertainties.

**Table 1:** Building construction information.

Construction	Information	U-value (W/m^2^·K)
Wall structure	Concrete masonry units with fiberglass R_SI_-1.9 (m^2^-K/W) and face brick	0.363
Floor	Slab-on-grade	0.104
Roof structure	Metal deck with polyisocyanurate (R_SI_-3.17) and ethylene propylene diene monomer	0.311
Windows	Double-pane clear glazing with aluminium frame	2.760

The RTU is as the main HVAC system for FRP-2. The direct expansion (DX) cooling coil and heating coil are installed in RTU. DX cooling coil consumes electrical energy and heating coil connects to the gas furnace. Each of the 10 conditioned zones has its own VAV box for reheating. The capacity of the RTU is 44 kW (12.5 ton) with a 9.6 energy efficiency rating.

### Test settings

This study included heating and cooling season tests. There were 4 different test settings for the heating season—baseline, free-floating, night set-back, and pre-heating. The cooling season test included the following three test settings: baseline, free-floating, and night set-back. Each test period lasted around 1 week.

For the baseline test, setpoint temperature was constant throughout the day. The HVAC system was always switched on and operated to meet minimum VAV airflow when heating and cooling load was absent from the test building.

For the night set-back and pre-heating tests, the three HVAC system operation hours included occupied hours, unoccupied hours, and pre-heating hours. HVAC system operation during occupied hours was the same as the baseline test. During unoccupied hours, the HVAC system was switched off (i.e., 0 m^3^/s airflow) when the indoor air temperature was between the 15.6 °C (heating set point temperature) and 29.4 °C (cooling set point temperature) and switched on when indoor air temperature was lower than the set point temperatures. During pre-heating hours, HVAC operation was identical to the occupied hour operation except for the heating and cooling set point temperatures. The information on the test periods is provided in Table 2.

**Table 2:** Test periods for heating and cooling season testing.

Test scenarios	Test period (DD/MM/YY)
**Heating season test**	
Free-floating test	23/02/21–28/02/21
Baseline test	04/03/21–10/03/21
Night set-back test	08/01/22–13/01/22
Pre-heating test	15/01/22–23/01/22
**Cooling season test**	
**Baseline test**	08/07/21–14/07/21
Free-floating test	20/07/21–25/07/21
Night set-back test	28/07/21–04/08/21

The test settings in the heating season are described in Table 3. For the pre-heating test, the HVAC system was switched off from 8 to 10 a.m. after the pre-heating from 5 to 8 a.m. The discharged air temperature set point for all scenarios, except for the free-floating test, was 13.9 °C.

**Table 3:** Test settings in heating season testing.

	Test setting		Test scenarios			
			Free-floating	Baseline	Night set-back	Pre-heating
Heating set point	Occupied		—	21 °C	21 °C	21 °C
	Unoccupied		—	—	15.6 °C	—
	Pre-heating		—	—	—	23.9 °C
Cooling set point	Occupied		—	24 °C	24 °C	24 °C
	Unoccupied		—	—	29.4 °C	—
	Pre-heating		—	—	—	29.4 °C
HVAC system operation	Gas furnace		Off	Off	Off	Off
	DX cooling		Off	On	On	On
	VAV box		Off	On	On	On
Operation hours	Turned on	Occupied	—	0 a.m.–12 a.m.	7 a.m.–10 p.m.	10 a.m.–5 a.m.
		Unoccupied	—	—	10 p.m.–7 a.m.	—
		Pre-heating	—	—	—	5 a.m.–8 a.m.
	Turned off		—	—	—	8 a.m.–10 a.m.

The test settings in the cooling season are described in Table 4. The discharged air temperature set point for all scenarios, except for the free-floating test, was 12.8 °C.

**Table 4:** Test settings in cooling season testing.

Test setting			Test scenarios		
			Free-floating	Baseline	Night set-back
Cooling set point	Occupied		—	24 °C	24 °C
	Unoccupied		—	—	29.4 °C
Heating set point	Occupied		—	21 °C	21 °C
	Unoccupied		—	—	15.6 °C
HVAC system operation	Gas furnace		Off	Off	Off
	DX cooling		Off	On	On
	VAV box		Off	On	On
Operation hours	Turned on	Occupied	—	0 a.m.–12 a.m.	7 a.m.–10 p.m.
		Unoccupied	—	—	10 p.m.–7 a.m.
	Turned off		—	—	—

Across all tests, some underlying test settings are as follows:

- Windows and doors closed
- Window blinds not used
- No internal heat gain from lighting, equipment, or people during test periods
- No outdoor air intake to the HVAC system
- Static pressure fixed at 249 Pa

## Data Records

**Fig. 2:**
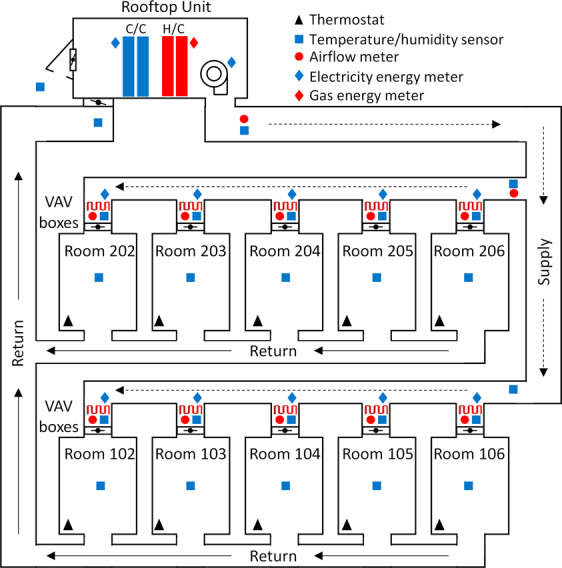
Building and HVAC system operation diagram.

The dataset is available at Figshare^25^. Figure 2 shows the sensor types and locations in the HVAC system. More than 500 sensors were installed in the test building, including zone temperature and relative humidity, supply and return air temperature, relative humidity, airflow rate, and power measurements. The field data were collected by the data logger (CR 3000) installed in FRP-2.

This dataset includes 16 comma-separated values (csv) files, 2 data description file, 7 weather data files, and 7 building data files, which means 1 weather data file and 1 building data file per test setting, as described in Table 5.

**Table 5:** Files of the test dataset.

Data file	Size (MB)	File description
**Free-floating test in the heating season**		
Weather_FF_Heating.csv	0.6	Weather data
Building_FF_Heating.csv	3.5	Building data
**Baseline test in the heating season**		
Weather_Base_Heating.csv	0.7	Weather data
Building_Base_Heating.csv	7.3	Building data
**Night set-back test in the heating season**		
Weather_SB_Heating.csv	0.6	Weather data
Building_SB_Heating.csv	5.4	Building data
**Pre-heating test in the heating season**		
Weather_Pre_Heating.csv	0.5	Weather data
Building_Pre_Heating.csv	4.7	Building data
**Free-floating test in the cooling season**		
Weather_FF_Cooling.csv	0.6	Weather data
Building_FF_Cooling.csv	3.5	Building data
**Baseline test in the cooling season**		
Weather_Base_Cooling.csv	0.7	Weather data
Building_Base_Cooling.csv	6.7	Building data
**Night set-back test in the cooling season**		
Weather_SB_Cooling.csv	0.8	Weather data
Building_SB_Cooling.csv	6.6	Building data

### Weather data

**Fig. 3:**
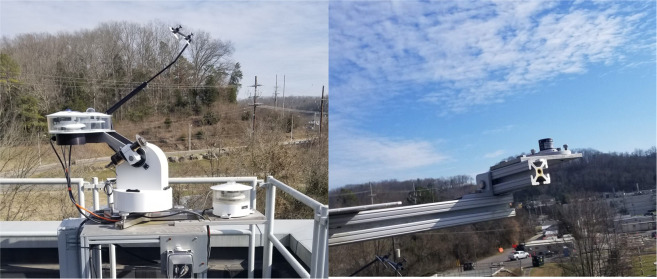
Weather station on the roof of FRP-2.

Figure 3 shows the dedicated weather station installed on the roof of FRP-2. Weather data were collected every 30 seconds and automatically generated data every minute and hour. The weather data variables and units are described in Table 6.

**Table 6:** Weather data variables and units.

Category	Data description	Unit	Data resolution
Weather	Outdoor air temperature	°C	1 min
	Outdoor air humidity	%	1 min
	Barometric pressure	Pa	1 min
	Direct solar radiation	W/m^2^	1 min
	Diffuse solar radiation	W/m^2^	1 min
	Global solar radiation	W/m^2^	1 min
	Wind speed	m/s	1 min
	Wind direction	°	1 min

### Building data

Building data were collected every 30 seconds and automatically generated data every minute and hour. Data variables and units are described in Table 7. The indoor conditions, air temperature, relative humidity, and set point temperature are included in the provided dataset. The supply and return air temperature, relative humidity, and set point temperature are also included. Energy consumption of the compressor, condenser, VAV reheating, and fan are included, along with RTU fan and VAV airflow rate.

**Table 7:** Building data variables and units.

Category	Data description	Unit	Data resolution
Indoor condition	Indoor air temperature	°C	1 min
	Indoor air relative humidity	%	1 min
Supply air	Supply air temperature (RTU)	°C	1 min
	Supply air temperature (VAV boxes)	°C	1 min
	Supply air relative humidity (RTU)	%	1 min
	Supply air relative humidity (VAV boxes)	%	1 min
Return air	Return air temperature	°C	1 min
	Return air relative humidity	%	1 min
Energy consumption	Compressor electric energy consumption	Wh	1 min
	Condenser electric energy consumption	Wh	1 min
	Re-heating coil electric energy consumption	Wh	1 min
	Fan electric energy consumption	Wh	1 min
Airflow rate	Airflow rate (RTU)	m^3^/s	1 min
	Airflow rate (VAV boxes)	m^3^/s	1 min

## Technical Validation

The field dataset was divided into two subsections for technical validation. For the reliability of the sensor reading, the team confirmed the installed sensor accuracy, and for validation of each field dataset per test setting, field data analysis was performed.

### Sensor accuracy

Table 8 describes the specifications of the installed sensors provided by the manufacturers. The quality of the dataset is reinforced by the sensors’ accuracy.

**Table 8:** Measurement variables and instrumentation used to collect data.

Measured data	Instrument	Range	Accuracy
**Weather data**			
Outdoor air temperature	Campbell Sci HC2S3-L	~−50–100 °C	±0.1 °C
Outdoor air humidity		~0%–100%	±0.8% at 23 °C
Barometric pressure	CS106 - Vaisala	500–1,100 hPa	±0.3 hPa at 20 °C
			±0.6 hPa (0 to 40 °C)
			±1.0 hPa (−20 to +45 °C)
			±1.5 hPa (−40 to +60 °C)
Direct solar radiation	Eppley sNIP (Normal Incidence Pyrheliometer)	Spectral range: ~250–3,500 nm, Output: 0–10 mV	±1% of reading (hourly)
Diffuse solar radiation	Eppley Model 8–48 (The Diffuse Pyranometer)	Spectral range: ~295–2,800 nm, Output: 0–10 mV	±2% of reading (hourly)
Global solar radiation	Eppley SPP (Standard Precision Pyranometer)	Spectral range: ~295–2,800 nm, Output: ~0–10 mV	±2% of reading (hourly)
Wind speed	Young Wind Monitor Model 05103	~0–100 m/s	±0.3 m/s or 1% of reading
Wind direction		~0°–360°	±3°
**Building data**			
Indoor air temperature	Campbell Sci HC2S3-L	~−50–100 °C	±0.1 °C
Supply air temperature			
Return air temperature			
Indoor air relative humidity		~0%–100%	±0.8% at 23 °C
Supply air relative humidity			
Return air relative humidity			
Compressor electric energy consumption	Continental Controls WNB-3D-240P	~0–72,000 W	±0.5% of reading
Condenser electric energy consumption			
Re-heating coil electric energy consumption			
Fan electric energy consumption			
Airflow rate (RTU fan)	Air monitor fan evaluators paired to DPT2500 Plus transmitters	~0–20.32 m/s	Fan evaluator: ±2%
			DPT2500 Plus transmitters: ±0.25% of natural span
Airflow rate (VAV boxes)	EF-x2000-T (EBTRON)	~0–15.24 m/s	±3% of reading

### Data analysis

A representative day was selected for each test scenario for field data validation purposes. Since test scenarios cannot be performed all at once, a representative day was selected based on the outdoor air temperature.

### Heating season testing

**Fig. 4:**
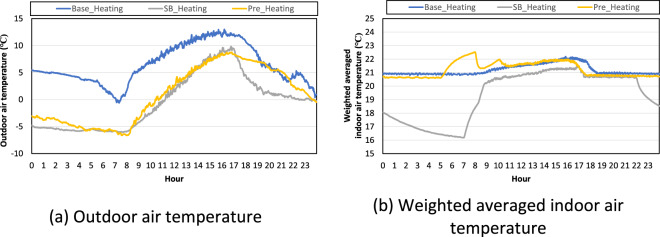
Outdoor air temperature and weighted averaged indoor air temperature in each test scenario in the heating season.

We selected representative days to validate the field dataset during the heating season. Figure 4a shows the outdoor air temperature pattern in each scenario. 6 March 2021, 8 January 2022, and 23 January 2022 were selected as representative days for the heating baseline test, night set-back test, and pre-heating test, respectively. Figure 4b shows the weighted averaged indoor air temperature pattern in each scenario. In the baseline test, the indoor air temperature was 21 °C. In the night set-back test, the indoor air temperature was around 21 °C during the occupied hours and it decreased during the unoccupied hours. In the pre-heating test, the heating set point temperature was 23.9 °C from 5 to 8 a.m., and the indoor air temperature increased until it reached 23.9 °C but decreased until it reached 21 °C after pre-heating.

**Fig. 5:**
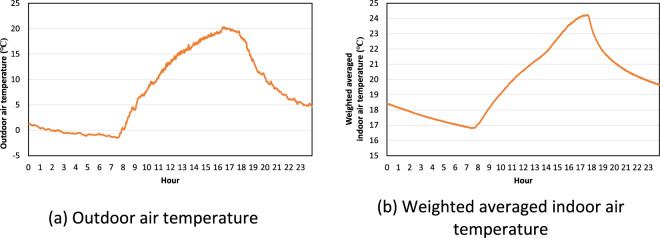
Outdoor air temperature and weighted averaged indoor air temperature in the free-floating test in the heating season.

During the free-floating test, the outdoor air temperature was higher than other heating test settings. Figure 5 shows the outdoor air temperature and weighted averaged indoor air temperature in free-floating test. 23 February 2021 was selected as a representative day—when temperature differences between minimum and maximum were the largest—to observe the weighted averaged indoor air temperature pattern. Because the HVAC system was switched off during the free-floating test, the weighted averaged indoor air temperature pattern was identical to the outdoor air temperature pattern.

**Fig. 6:**
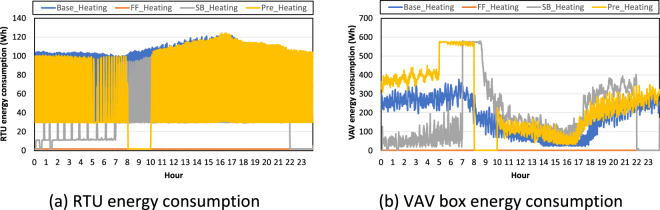
RTU and VAV box energy consumption in each test scenario in the heating season.

Figure 6a,b show the RTU and VAV box energy consumption in each test scenario. The RTU was on and off frequently during the heating season. During the baseline test, the RTU was switched on all day because the HVAC system was also switched on all day. The RTU was never switched on during the free-floating test. During the night-setback test, the RTU was switched on during the occupied and unoccupied hours when the heating load occurred because RTU energy consumption included the RTU supply fan. During the pre-heating test, the RTU was switched on all day, except from 8 to 10 a.m., when the HVAC system was switched off after the pre-heating from 5 to 8 a.m.

The VAV boxes consumed energy all day during the baseline test. They consumed more energy during the night when the heating load was higher. The VAV boxes were not switched on for the free-floating test. For the night set-back test, the VAV boxes were switched off from 10 p.m. to 12 a.m. after the occupied hour; however, the VAV boxes were switched on from 12 a.m. because of dropping indoor air temperatures. The VAV boxes consumed more energy than during the baseline test from 7 to 9 a.m. to increase the indoor air temperature to the 21 °C heating set point temperature. During the pre-heating test, the VAV boxes consumed more energy from 5 to 8 a.m. because of the higher heating set point temperature (i.e., increased from 21 °C to 23.9 °C) for pre-heating purposes. The VAV boxes were switched off from 8 to 10 a.m. because the HVAC system was also switched off during these hours.

### Cooling season testing

**Fig. 7:**
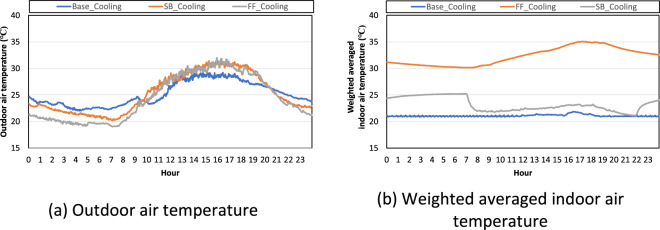
Outdoor air temperature and weighted averaged indoor air temperature in each test scenario during the cooling season.

We selected representative days to validate the field dataset during the cooling season. Figure 7a shows the outdoor air temperature pattern in each scenario. 9 July 2021, 21 July 2021, and 31 July 2021 were selected as representative days for the cooling baseline test, free-floating test, and night set-back test, respectively. Figure 7b shows the weighted averaged indoor air temperature pattern in each scenario. In the baseline test, the indoor air temperature was almost constantly recorded at 21 °C. In the night set-back test, the indoor air temperature was around 22 °C during the occupied hours and 25 °C during the unoccupied hours. In the free-floating test, the indoor air temperature changed depending on the outdoor air temperature.

**Fig. 8:**
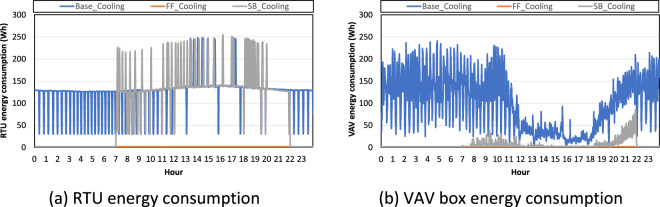
RTU and VAV box energy consumption in each test scenario in the cooling season.

Figure 8a,b show the RTU and VAV box energy consumption from each test scenario. The HVAC system was switched off for the free-floating test, which means there was no RTU or VAV box energy consumption. For the night set-back test, the HVAC system was switched on during occupied hours and when the indoor air temperature was higher than 29.4 °C during unoccupied hours. Since indoor air temperature of the night set-back test was around 25 °C during unoccupied hours (Fig. 7b), the HVAC system was switched on when the indoor air temperature was higher than 25 °C during unoccupied hours. Since indoor air temperature was never higher than 25 °C during unoccupied hours, the HVAC system was not switched on until 7 a.m. During the baseline test, the HVAC system was switched on all day, and the RTU and VAV boxes were on all day. Since outdoor air temperature during day was higher than during the night, the VAV boxes consumed more energy during the day.

**Fig. 9:**
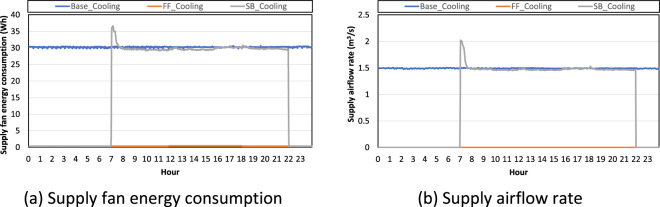
Supply fan energy consumption and supply airflow rate in each test scenario in the cooling season.

To explain why indoor air temperature was around 21 °C as opposed to 24 °C, which is the cooling set point temperature (Fig. 7b), We analysed the fan energy consumption supply airflow rate (Fig. 9a,b). Even if the cooling set point temperature was 24 °C for the cooling season test, the indoor air temperature was around 21 °C in both the baseline and night set-back tests. This is caused by the VAV box operation. Even when the indoor air temperature was within a comfortable range which is between the heating and cooling set point temperatures, the VAV box operated with a minimum airflow rate. Consequently, all conditioned zones received supply air with minimum airflow rates when the HVAC system was switched on. As an underlying test setting, the RTU supply air set point temperature was 12.7 °C; however, this set point was too low when the indoor air temperature is in a comfortable range. To increase the supply air temperature, the supply air was reheated by the VAV box. Thus, the indoor air temperatures of the baseline and night set-back tests were closer to the heating set point temperature (21 °C) and not the cooling set point temperature (24 °C). Because the VAV box operates to reheat supply air with minimal airflow rates, the VAV box consumes more energy at night (Fig. 8b). For these reasons, the RTU supply airflow rate was constant in the baseline tests. Since the RTU supply airflow rate was the same as the supply airflow rate from the 10 VAV boxes with no duct leakage, the RTU supply airflow rate was constant because each VAV box requested a constant minimal airflow rate.

## Data Availability

No code was used in the generation of this data. No code is required to access or analyse this dataset.
